# The Interplay Between Environment and Drug Effects: Decoding the Ecocebo Phenomenon with Virtual Technologies

**DOI:** 10.3390/s25175268

**Published:** 2025-08-24

**Authors:** Thomas Zandonai, Cristiano Chiamulera

**Affiliations:** 1 Department of Pharmacology, Pediatrics, and Organic Chemistry, Miguel Hernández University of Elche, Sant Joan, 03550 Alicante, Spain; 2 Addiction Science Laboratory, Department of Psychology and Cognitive Science, University of Trento, 38068 Rovereto, Italy; 3 Department of Diagnostics and Public Health, University of Verona, 37134 Verona, Italy

**Keywords:** Ecocebo, environment, virtual reality, pharmacotherapy, placebo

## Abstract

In this perspective article, we introduce Ecocebo as a novel concept describing the modulatory effects of physical environments, whether natural or built, on drug effect. Positioned as a spatial component of the placebo effect, Ecocebo is grounded in evidence-based design principles and proposes that environmental features such as natural light, greenery, spatial geometry, and calming esthetics can significantly influence sensory, emotional, and cognitive processes. These environmental factors may enhance or modify pharmacological responses, especially for analgesics, anxiolytics, and antidepressants. We highlighted how exposure to restorative spaces can reduce pain perception, stress, and the need for medication, paralleling findings in placebo research where contextual and sensory cues influence brain regions linked to emotion and pain regulation. We propose virtual reality (VR) as the most suitable methodological tool to study Ecocebo in controlled and ecologically valid settings. VR allows for the precise manipulation of spatial features and real-time monitoring of physiological and psychological responses. We also propose integrating VR with neuromodulation techniques to investigate brain–environment–drug interactions. Finally, we addressed key methodological challenges such as defining control conditions and standardizing the measurement of presence. This perspective opens new directions for the integration of non-pharmacological and pharmacological interventions and personalized therapeutic environments to optimize clinical outcomes.

## 1. Ecocebo: The Physical Component of Placebo Response

Evidence-based design (EBD) research demonstrates that environmental properties influence autonomic, sensorial, perceptual, and cognitive systems, affecting emotion, mood, and behaviour [1,2]. Features like natural light, greenery, and calming esthetics correlate with reduced pain, stress, and depressive symptoms [3]. Environmental cues also affect perception, memory, attention, pleasure, and esthetic judgement [4]. A new term recently proposed by Chiamulera et al. [5], Ecocebo, (the fusion between the prefix ‘eco-’, environment, and the suffix ‘-cebo’, referring to placebo), describes the modulatory effect of physical environments (natural or built) on drug action, a concept supported by EBD. Similarly to placebo effects, Ecocebo addresses how specific, non-pharmacological, spatial features can enhance or alter pharmacological responses, particularly in the case of analgesics, anxiolytics, and antidepressants [5].

### 1.1. The Spatial Context Component of Placebo

Ecocebo is a component of the contextual factors of placebo effect. Placebo research has shown that contextual factors (e.g., healthcare setting) induce unconscious and conscious effects—at emotional, cognitive, and sensorial level—that may contribute to therapeutic, including the pharmacological, outcomes [6]. Moreover, learning processes such as conditioning, reward, and expectation have been shown to underlie the placebo effect as it develops and is maintained over time. Placebo effects do indeed induce changes in brain areas involved in the conditioning and regulation of nociceptive, emotional, and cognitive processing integration [7]. Placebo-induced changes to these brain areas, circuits, and processes may thus be correlated to reduction in pain and anxiety and improved mood and cognitive functions [8]. Similarly to the placebo effect, specific components of the spatial context may interact with the pharmacological effect of drugs, leading to an enhanced pharmacological response.

### 1.2. The Interaction Between Environmental Space and Response to Medications

The pioneer study by Roger S. Ulrich in *Science* magazine [9] described the effects of hospital room features on post-surgical outcomes. Patients assigned to rooms with window access to natural scenery and light showed a better recovery compared to patients assigned to rooms facing brick walls. These patients took less strong medication (e.g., narcotics) and only mild (e.g., aspirin or acetaminophen) analgesics, with a significant difference to the group in wall-view rooms [9]. The then-defined EBD activities generated empirical data on those physical features of the environmental context that may act as modulators of pain as well as anxiety and mood.

Natural light exposure represents a critical environmental factor. Walch et al. [10] found that spinal surgery patients exposed to high-intensity sunlight required 22% less pain medication per hour compared to those in low-light conditions. Donovan’s retrospective study revealed that patients recovering from joint surgery who lived in greener neighbourhoods exhibited significantly reduced opioid use over a 12-month follow-up period, suggesting sustained environmental effects on pain management. Artwork selections in healthcare interiors also influence analgesic requirements. Patients typically prefer nature scenes dominated by blues and greens—colours shown to elicit more pleasure and less arousal than reds and yellows [11].

This research highlights how environmental context functions as a significant modulator of pain perception and medication efficacy, offering promising avenues for complementary non-pharmacological interventions that could reduce medication requirements while improving patient outcomes and experience. Its practical applications include (i) tailoring environments to individual patients to enhance drug treatment efficacy; (ii) hospital designs incorporating natural light, green spaces, and calming esthetics; and (iii) smart homes with personalized treatment-monitoring capabilities.

### 1.3. Open Questions Regarding the ‘What’ and ‘How’ of Investigating Ecocebo

Firstly, more research is needed to establish the relationship between the type of space design, drug action, and underlying processes and mechanisms. What are the psychobiological effects of the exposure to those spatial features of the environment that contribute to a modified (Ecocebo) drug response? Which specific physical features must be present in the spatial context “to act as Ecocebo”?

Secondly, despite the availability of different methodological approaches, which experimental strategies and technological approaches may help to provide a translational link between lab studies and the complexity of the ecological investigations in different environmental conditions? How can the Ecocebo response be studied with a technology with translational validity?

## 2. The Psychobiological Effects Induced by Environmental Features

As mentioned before, the environment may induce changes in affect and emotion [1]. Investigations in healthcare and rehabilitation centres showed that a single-bed room, access to light, and views of nature improve affective states and reduce stress and anxiety. Exposure to natural daylight through wide windows that allow illumination of the space and access to a view of nature appear to have consistent effects. On the other hand, low lighting levels in the bedroom were associated with a negative affect on mood.

Research on the geometry and form of the space suggests the importance of facility size. The layout of a room may impact on the duration of the recovery period in hospital [12], whereas the density of the space and aspect ratio (e.g., high density and short depth, larger horizontal space and high ceilings) have been shown to induce stress. Environmental disorder is linked to anxiety [13], whereas clear landmarks and cues provide orientation [12]; the lack of these landmarks triggers stress and anxiety.

It could therefore be considered that the Ecocebo effect may interact with the pharmacodynamic mechanisms of the drug, as well as, concomitantly, directly affecting the pathophysiological mechanisms underlying the symptoms and the disorders, for instance, by increasing the pain threshold. In the latter case, we could suggest that the Ecocebo phenomenon is not only an ‘enhancement/co-adjuvant’ of the pharmacological response, but also a direct placebo response per se.

### Emotional and Neuropsychological Impact of Interior Design Elements

The curvature of room walls, compared to spaces with corners, induces a feeling of pleasure, and is correlated with positive emotions and mental activation (e.g., [14]). Analysis of the underlying mechanisms of this has revealed the distinct activity of brain regions, such as the anterior cingulate cortex, responsible for integrating affective and cognitive processes, motor possibilities, and exploratory activation. Vartanian and colleagues [15] showed how people prefer rounded contours, and this preference arises precisely in the anterior cingulate cortex, where pleasure is associated with cognitive awareness. Furniture with sharp edges induces the activation of the amygdala, a brain area responsible for recognizing stimuli with emotional value [16].

The materiality of interiors (e.g., wood texture) and colours affect emotional and affective responses, as shown by changes in neurovegetative responses. For instance, red-painted rooms increase stress. Other interventions have been shown to affect mood; the predominance of the colour blue may have a depressive effect. A home-like environment favours a sense of privacy and familiarity that may positively modulate affective responses. As in residential units, the inclusion of decorative elements that evoke domestic environments [17] or that are associated with past experiences and memories (e.g., old-fashioned gym interiors) may contribute to achieving a balance between familiarity and calm, and novelty and stimulation.

There are, however, two issues that deserve further investigation, respectively, environmental complexity and subjective psychobiological changes. Although research has been primarily focused on psychobiological dependent variables (i.e., stress, depression, pain, cognitive disorders), there are other outcomes which would need to be investigated to form an index of treatment efficiency, such as the subjective and phenomenological response of individuals and patients exposed to different space conditions [18]. Esthetic responses to spaces have been widely investigated, and this is the prevalent conceptual framework for investigations in neuro-architecture [19].

Furthermore, not only materiality, form, and geometry, but also complexity (information and richness), organization (order, symmetry, redundancy), style, naturalness, and beauty should be examined in relation to drug treatment [4]. More evidence is needed in order to identify those variables that depend on the individual’s state (e.g., healthy vs. patient, male vs. female, young vs. old), disease stage, disease gravity, and preferences [17,20]. For instance, colour features (e.g., is blue depressive or calming? Is red stimulating or anxiogenic?) and space ratio parameters have been shown to be related to an individual’s state and/or traits.

## 3. Which Experimental Strategy and Technological Approaches Should Be Used to Study Ecocebo?

The configurational pattern of the physical space under investigation, whether a set of variables to be correlated or an independent variable to be causally linked, in relation to drug response presents an intrinsic complexity that is difficult to make constant, reproducible, and logistically feasible. Pre-existing natural or built environments are not designed a priori for research purposes, while ad hoc experimental settings may be short-lived and limited to a single study. Thus, a translational approach is needed in between ecological/field studies and video/image exposure tests with the aim to increase both the back and forward translational validity of mimicking environmental complexity and the parametric identification of key elements, respectively.

### 3.1. Advancing Experimental and Clinical Neuroscience Through Virtual Reality Technologies

Virtual reality (VR) is a feasible methodological approach for modelling the parametric features of the environment in a laboratory setting [21,22]. VR allows the user to the interact with, and become immersed in, a computer-generated environment in a naturalistic-like way. The key features that characterize VR are (i), immersion, i.e., the extent to which the user perceives him/herself in the virtual environment rather than the real world; (ii) sense of presence, i.e., the subjective experience of the user as being in the virtual world; and (iii) the possibility to interact with the computer-generated environment [23].

VR has some advantages over traditional approaches. Firstly, VR does indeed have a high level of ecological validity because of the sensorimotor interaction between the user and the virtual environment, allowing the transfer of skills from the virtual to the real world. Secondly, VR has the advantage of providing quantitative outcome measures under a controlled experimental setting. For instance, Presti et al. [24] described a VR parametric model that generated virtual architectural designs with progressive variations in room size, proportions, and colours that significantly correlated to emotional states. Lastly, VR allows subjects to be exposed in a safe and controlled environment [24]. For instance, VR has been extensively used in research and therapy for post-traumatic stress disorders with exposure to the virtual retrieval of traumatic memories [25,26]. Similarly, several drug cue- and context-reactivity studies have been conducted in order to characterize conditions at risk of relapse, as well as anti-craving virtual therapeutic interventions [27]. In drug addiction research, we demonstrated that VR post-retrieval extinction following smoking-related memory reactivation was significantly more effective than standard extinction procedures. These findings suggest that the immersive and contextually rich environment provided by VR enhances the reconsolidation memory window’s vulnerability, leading to the attenuation of cue-induced craving and potentially greater long-term relapse prevention in smokers [28].

In addition, we explored the potential anti-craving effects of environmental enrichment (EE). EE consists of a complex spatial context configuration that stimulates motor, sensory, and cognitive processes inside animal housing cages. The main challenges involve translating EE settings to human studies and clinical practice, considering that reproducing protocols of similar complexity remains difficult. By using VR, we were able to reproduce the same sensorimotor stimulations and engagements shown in animal studies. We reported that experience with virtual EE was able to reduce craving for palatable food in healthy volunteers and for cigarettes in smokers [29,30,31]. We recreated enrichment conditions similar to laboratory animal studies by applying specific tasks/variables (e.g., maze navigation, motor coordination) to be correlated with psychometric and physiological metrics in humans. Another approach, as recently proposed by Barillot et al. [32], is to use more complex, multidimensional, and multimodal environmental enrichment parameters with higher translational value.

### 3.2. Sense of Presence and Ecological Validity in VR: Toward Better Standards for Experimental and Therapeutic Research

VR has been used to compare the effects of a virtual simulation of standard hospital vs. sea-view rooms in migraine pain patients [33]. The immersion in the VR sea-view room was able to reduce pain reports compared to control conditions, an effect correlated to changes in brain activation. In a pioneering study, Valtchanov and Ellard [34] previously found that virtual green environments correlated with reduced prefrontal cortex activity, suggesting benefits for patients with affective disorders. Nowadays, there has been increasing scholarly interest in employing VR to investigate the effects of nature exposure and related experiences, a trend supported by substantial evidence demonstrating the beneficial impact of forest environments [35] and mountain therapies [36]. Given that the development of nature-based experiences in VR entails greater complexity than that of built environments, current research is examining the impact of the use of technological factors (computer-interaction, e.g., [37]) in special populations (e.g., the elderly; [38]) and on different psychological and physiological assessments (for a systematic review see, [39]).

In spite of the widespread adoption of VR, greater effort is required to temper the uncritical enthusiasm that often accompanies its use. While VR represents a relatively cost-effective and accessible technology, the establishment of consensus-based criteria for study design protocols remains necessary, particularly with respect to the definition of appropriate control conditions and comparison groups in elderly populations [37]. The lack of standardized control conditions compromises the reproducibility and generalizability of findings across studies. The VR-CORE framework, a consensus-driven model for conducting clinical trials involving VR, emphasizes the need for clear definitions of control groups, standardization of intervention protocols, and methodological rigour. This framework advocates for the implementation of phased trial designs and consistent outcome measures, thereby promoting the comparability of results across diverse experimental settings [40].

Consensus is also needed for the peculiar VR-dependent variable that is the sense of presence (see [41]). At present, sense of presence is measured as a subjective and/or objective measure, important for the assessment of the effects induced by both the elements of the virtual scenario and the interaction with the technological features of the simulation [42]. Indeed, higher sense of presence may be a relevant index of the validity of VR as a model of ecological and naturalistic complexity, given its computer interaction features [43]. A growing body of research underscores the critical role of sense of presence in VR, as it influences both emotional engagement and ecological validity [44]. Recent studies have demonstrated that objective physiological measures can be effectively used to quantify presence. When combined with validated subjective instruments, such as questionnaires, these biometric indicators offer a multidimensional framework for evaluating user experience and tailoring VR interventions to individual responsiveness [45].

As mentioned above, exposure to virtual nature has been shown to evoke positive emotional states, reduce stress and anxiety, enhance relaxation, and have a restorative effect. Such benefits are closely linked to a heightened sense of presence, as nature-based virtual environments often elicit a stronger feeling of ‘being there’ compared to built environments. These effects are particularly beneficial for populations with limited physical access to natural settings, where virtual nature can serve as a meaningful psychological substitute for real-world exposure [46]. Finally, to fully realize the potential of VR, it is imperative to extend research to diverse user populations. This includes elderly individuals, who may experience cognitive or sensory changes, as well as clinical groups such as those with depression, anxiety, or post-traumatic stress disorder [47]. Studies suggest that immersive VR experiences can foster positive affective responses, improve mood, and support emotional regulation in older adults, thereby highlighting the promise of VR as a non-pharmacological tool for mental health support [48].

Finally, recent studies have shown that the metaverse, which can be defined as an immersive, persistent, and interoperable digital ecosystem integrating multiple technologies (including VR, augmented reality (AR), artificial intelligence, and digital identities) [49], extends the potential of traditional VR by providing social, continuous, and highly customizable virtual spaces. Within these environments, patients and mental health professionals can interact through avatars and engage in adaptive therapeutic protocols with potential improvement of the efficacy of drug response [50]. The social and collaborative dimension of the metaverse facilitates the overcoming of geographical and logistical barriers and may potentially contribute to reducing the stigma associated with psychiatric and substance-use disorders, thereby enhancing accessibility, inclusivity, and user engagement [49,50]. Nevertheless, several critical issues remain to be addressed, including the protection of privacy and security in the management of sensitive clinical data, as well as the risk of emerging forms of behavioural addiction within immersive environments [49,51].

## 4. Future Developments

Our recommendation to prioritize the use and further development of VR for studying the Ecocebo phenomenon is grounded in its strong ecological validity for mimicking environmental complexity. Nevertheless, integrating VR technology with additional neurophysiological approaches may yield further insights into the correlates of altered drug responses.

Neuroimaging (e.g., fMRI, PET, NIRS) and neurophysiological (e.g., EEG, MEG) techniques have been used alongside VR in order to investigate the brain activity correlates of virtual simulation effects (e.g., exposure to natural or built context and stimuli) on different psychological responses [52]. However, neuroimaging and neurophysiological techniques combined with VR may restrict participants’ freedom of interaction—such as head movements—within the virtual simulation, thereby reducing the feasibility of psychological and physiological assessments. In order to address these limitations, the use of VR in real lab spaces (for instance, in the experimental lab walls called CAVE [53]; portable MR (e.g., [54]); or EEG (e.g., [55]) has presented a feasible solution.

Particularly attractive is the feasibility of employing non-invasive, low-cost sensors for physiological measures (e.g., skin conductance, SC; heart rate variability, HRV) [56]. The integration of biometric monitoring in VR for assessing emotional responses represents a promising methodological opportunity, particularly in clinical contexts involving patients with cognitive or motor impairments undergoing pharmacological treatments [57]. Recent advances in wearable technologies have made it increasingly feasible to collect high-quality physiological data in real time without compromising user comfort or immersion [58,59].

Furthermore, using machine learning techniques to classify emotional states from multimodal biosignals can enhance the sensitivity of emotion detection, enabling adaptive VR scenarios tailored to individual affective responses [60]. Such an approach has particular relevance for populations with limited verbal communication or cognitive deficits, offering objective markers of emotional engagement and discomfort. In the context of neuropsychopharmacology, this integration can capture subtle drug response changes in the interactive settings.

VR sessions can be designed to synchronize visual/auditory stimuli with non-invasive brain stimulations (NIBS). The integration of VR with NIBS techniques, such as transcranial magnetic stimulation (TMS) and transcranial direct current stimulation (tDCS), may represent an approach in neurorehabilitation, cognitive enhancement, and mental health interventions. This emerging association may enable the alignment of brain stimulation with immersive VR experiences, thereby allowing for a better understanding of brain processes and mechanisms underlying the Ecocebo effect. Brain stimulation can be precisely coordinated with virtual simulations by timing electrical stimulation to coincide with specific visual or auditory cues [61]. The synchronization of visual and auditory stimuli within VR environments with NIBS may allow for precise modulation of neural circuits, potentially amplifying the outcome of the interaction between environment X and drug effects [62].

One innovative protocol combined VR, EEG, and TMS to investigate the neural dynamics associated with the experience of awe. Participants were immersed in VR scenarios specifically designed to evoke intense emotional responses, while EEG recorded real-time brain activity. TMS was then used to assess changes in cortical excitability and connectivity. This approach highlights the potential of VR–TMS integration to examine complex emotional and cognitive processes in a highly controlled and immersive setting [63]. Similarly, combining repetitive TMS (rTMS) with VR training in patients recovering from stroke has shown that the simultaneous application of rTMS and VR leads to greater improvements in sensorimotor and cognitive functioning than either modality alone [64].

Real-time brain monitoring allows for the dynamic adjustment of stimulation parameters throughout the session. This creates closed-loop system in which NIBS continuously responds to both neural signals and user performance, optimizing therapeutic impact. The association between VR, NIBS, advanced neuroimaging, and neurophysiological techniques may therefore enable a multimodal assessment of the complex and multidimensional effects of virtual exposure across both time and space. With this approach, the phenomenon of Ecocebo may be investigated and characterized for its effects at the psychological and brain levels on drug response.

## Data Availability

Data are contained within the article.

## Abbreviations

The following abbreviations are used in this manuscript:

EBD	evidence-based design
EE	environmental enrichment
EEG	electroencephalogram
HRV	heart rate variability
NIBS	non-invasive brain stimulations
SC	skin conductance
VR	virtual reality
